# Factors associated with repeat induced abortion in Kenya

**DOI:** 10.1186/s12889-015-2400-3

**Published:** 2015-10-12

**Authors:** Beatrice W. Maina, Michael M. Mutua, Estelle M. Sidze

**Affiliations:** Population Dynamics and Reproductive Health Program, African Population and Health Research Center (APHRC), P.O. Box 10787–00100, Nairobi, Kenya

**Keywords:** Repeat abortion, Unintended pregnancy, Contraceptive use, Kenya

## Abstract

**Background:**

Over six million induced abortions were reported in Africa in 2008 with over two million induced abortions occurring in Eastern Africa. Although a significant proportion of women in the region procure more than one abortion during their reproductive period, there is a dearth of research on factors associated with repeat abortion.

**Methods:**

Data for this study come from the Magnitude and Incidence of Unsafe Abortion Study conducted by the African Population and Health Research Center in Kenya in 2012. The study used a nationally-representative sample of 350 facilities (level II to level VI) that offer post-abortion services for complications following induced and spontaneous abortions. A prospective morbidity survey tool was used by health providers in 328 facilities to collect information on socio-demographic charateristics, reproductive health history and contraceptive use at conception for all patients presenting for post-abortion services. Our analysis is based on data recorded on 769 women who were classified as having had an induced abortion.

**Results:**

About 16 % of women seeking post abortion services for an induced abortion reported to have had a previous induced abortion. Being separated or divorced or widowed, having no education, having unwanted pregnancy, having 1–2 prior births and using traditional methods of contraception were associated with a higher likelihood of a repeat induced abortion.

**Conclusions:**

The findings point to the need to address the reasons why women with first time induced abortion do not have the necessary information to prevent unintended pregnancies and further induced abortions. Possible explanations linked to the quality of post-abortion family planning and coverage of long-acting methods should be explored.

## Background

Over six million induced abortions were reported in Africa in 2008 [1] with over two million occurring in Eastern Africa^1^ despite abortion being largely illegal in the region. The relatively high unsafe abortion rate in Africa (28 unsafe abortions per 1000 women aged 15–44) was only second to the Latin America and the Caribbean at 31 unsafe abortions per 1000 women aged 15–44 in 2008 [1]. Sub-regionally, Eastern and Middle Africa topped the world at a rate of 36 unsafe abortions per 1000 women aged 15–44 years. About 14 % of maternal deaths in Africa are attributed to unsafe abortion [1] and almost two million women in Africa are hospitalized annually due to complications resulting from unsafe abortions [2]. Other adverse effects associated with abortion include severe hemorrhage, infections, trauma, and renal failure [3, 4].

Studies indicate that despite the adverse effects on health, a significant proportion of women procure more than one abortion during their reproductive lifetime [5–9]. In Sudan for instance, a study in five hospitals indicated that over 40 % of women seeking treatment for complications of unsafe abortion had at least one previous unsafe abortion [8]. Another study in public and private facilities in Ethiopia indicated that among women seeking abortion-related services, the incidence of repeat abortion was 30 % [9].

Despite the high prevalence of repeat induced abortions observed in African settings, there is a dearth of research on factors associated with repeat induced abortions. Studies on abortion have mainly focused on factors associated with induced abortions while considering previous induced abortions as one of the risk factors [8–10]. Extant studies from elsewhere in the world suggest that age, parity and contraceptive use are associated with the risk of repeat abortion [5, 6, 11, 12]. A study carried out in Nepal showed that the risk of repeat induced abortion increases linearly with age [5]. Another study carried in the United States of America also showed that older women were significantly more likely to have repeated elective abortions than teenagers [12]. However, a study in Finland found that young women were more likely to seek a repeat induced abortion within 5 years compared to older women [11]. As regards the effect of parity, a study in the United States of America found that women having repeat abortions were not only likely to have had prior births, but were more likely to have had three or more prior births [13]. Similar findings were found in the Nepalese study [5]. On the effect of contraception, a number of studies have shown that most women who have induced abortions were non-users of contraceptive methods or were using less efficient contraceptive methods during the month they became pregnant [5, 10, 11, 13–18]. Similarly, women not using contraception were more likely to seek another abortion compared to those on contraception [11].

This paper provides further evidence on factors associated with repeat abortion in African settings in particular. The study focuses on Kenya, a country in Eastern Africa where the prevalence of unsafe abortions is relatively high. Kenya had an estimated 1.6 million live births in 2012 [7]. An estimated 119,912 cases of induced abortions were treated at heath facilities during the same year [7]. It is estimated that about 13 % of maternal deaths in Kenya are as a result of unsafe abortions [19]. Kenya is still lagging behind in regard to the Millennium Development Goal (MDG) 5 on improving maternal health by reducing maternal mortality and morbidity [19]. According to the 2008–09 Kenya Demographic and Health Survey (KDHS), the maternal mortality rate in Kenya was 488 deaths per 100,000 live births [20], an increase from 412 deaths per 100,000 live births recorded in 2003 [21]. Prevention of repeat induced abortions is therefore key in attainment of MDG 5 by reducing maternal related morbidities and mortalities due to unsafe abortions.

## Methods

### Data

Data used in this paper were collected as part of the Magnitude and Incidence of Unsafe Abortion Project conducted by the African Population and Health Research Center (APHRC) in 2012. The project was carried out in partnership with IPAS, the Guttmacher Institute and the Kenyan Ministry of Health and other local partners [7].

### Procedures

Data were collected in public and private health facilities between March and September 2012. A stratified random sampling strategy was used to select participating health facilities. The stratification was done by health facility level (Level II to Level VI), ownership type (public or private/non-governmental) and five larger geographical regions (Nairobi and Central, Eastern, Coast and North-Eastern, Nyanza and Western, and Rift Valley). The sampling frame included all facilities from Level II to Level VI that had capacity to offer post abortion services as of January 31, 2012. This included 2838 facilities. Participating health facilities were selected using the following sampling fractions: Level II (0.05–0.10), Level III (0.08–0.15), Level IV (0.18–0.36), Level V (all facilities on the list) and Level VI (all facilities on the list). A total of 350 facilities were sampled for the study.

Three survey tools were used for data collection: a Health Professionals Survey (HPS), a Health Facilities Survey (HFS), and a Prospective Morbidity Survey (PMS). The HPS sought providers’ perspectives on access to and provision of post-abortion care services in Kenya. The HFS collected information on the numbers of women seeking post-abortion services, the availability of trained staff and the type of services offered at facilities. The PMS, which is used for this study, collected prospective data on all patients presenting for post abortion services or termination of pregnancies during a 30-day period. The PMS data were collected by trained facility-based health providers. Relevant information for this study includes: patients’ socio-demographic characteristics, reproductive health history and contraceptive use.

### Study sample

The PMS collected information on patients in 328 facilities. Two facilities with poor quality data were dropped. This study uses information collected in 326 facilities on 2631 women. These were actual women observed during the 30-day period.

### Measurements

The dependent variable is “repeat induced abortion”, a binary indicator of whether the woman had an induced abortion prior to the current one. Induced abortion even at facility level is illegal in Kenya unless, in the opinion of a trained health professional, there is need for emergency treatment, or the health of the mother is in danger, or if permitted by any other written law [22]. Induced abortions in this study refer to abortions induced in an unsafe manner outside health facilities and for which women were receiving post abortion care in health facilities after complications occurred. Induced abortions were identified using multiple components: report by patient as part of normal medical history, doctor’s examination and later at analysis, where a number of responses and findings from the physician records were compared to identify any abortions reported as spontaneous while they were actually induced. The physicians collected data for other observations and examinations such as evidence of lacerations on the vaginal wall, presence of foreign materials and any other evidence of any methods of induction.

**Fig. 1:**
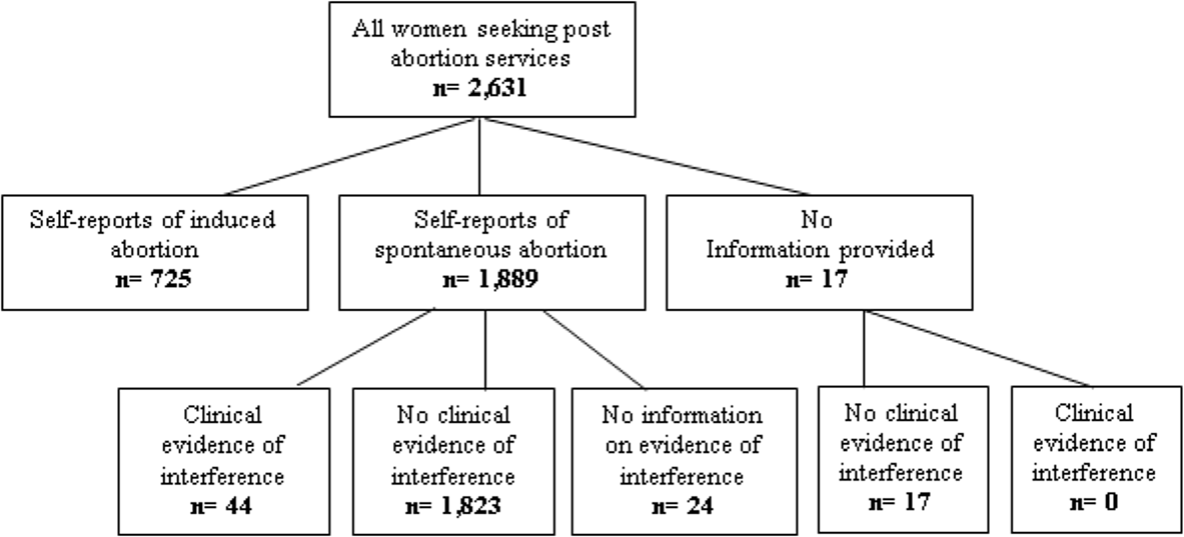
Classification of post-abortion cases

As depicted in Fig. 1, from the total of 2631 women presented for post-abortion services, 725 reported to have induced the abortion, 1889 reported to have had a spontaneous abortion, and 17 women did not provide any information. Forty four (44) cases that were reported as spontaneous were grouped as induced abortions after medical examination. Clinical evidence included: signs of instrumentation in the uterus or cervix, sepsis caused by introduction of foreign substances, and evidence of misoprostol. It is important to note that there is a possibility that some women might have denied having had their pregnancy interfered with and at the same time had no evidence of interference at clinical examination. Such cases, if they existed, were classified as spontaneous abortions. A total of 769 cases were considered as induced abortion. Cases of repeat induced abortion were determined by women self-reports of prior induced abortion. It is also important to note that not all women report their prior abortions, and hence the number of prior abortions can only be indicated as a minimum estimate.

### Data management and analysis

Data were captured using paper forms by trained facility-based health providers, entered using CSpro, and exported to STATA Version 12.1 for consistency checks and analysis. Both bivariate and multivariable analyses (binary logistic regressions) are presented. The multivariable results presented here were generated using logistic regression implemented within the Stata’s *svy set* function in order to control for the survey design in our analysis as well as to control for the clustering effect due to similarity of women seeking care in the same facility than those seeking in different facilities. Independent variables include: age, place of residence, marital status, level of education attained, number of live births, pregnancy wantedness, gestational age, and contraceptive use at the time of conception.

### Ethical considerations and data access

The study protocol was approved by the ethical review boards of the Kenya Medical Research Institute (KEMRI), the University of Nairobi/Kenyatta National Hospital, Moi University Teaching and Referral Hospital (Kenya), and Aga Khan University (Kenya). Clear guidelines to comply with ethical considerations were used during data collection. Verbal consents were obtained from all women presenting for PAC. Deidentification of records was done before data analysis to ensure that all data collected on a woman, provider or facility could not be traced back to the source. The African Population and Health Research Centre (APHRC) in Nairobi, Kenya owns the Magnitude and Incidence of Unsafe Abortion study data and is responsible for its storage and use. For external users, these data are publicly available online via the APHRC Microdata portal <http://aphrc.org/catalog/microdata/index.php/catalog/39> upon registration and completion of an online data request form. More details about the survey are available elsewhere [7].

## Results

Table 1 presents the sociodemographic characteristics of the women in the sample by history of induced abortion. About 16 % of women who presented at the facilities for post abortion services after an induced abortion reported to have had a previous induced abortion. Significant differences can be noted by age and marital status. The proportion of women who had had a previous abortion was highest among those aged 20–24 years while the proportion of women having a first-time induced abortion was highest among those aged 10–19 years (93 %). Although the proportion of women who had a first-time induced abortion was higher among women who were never married (88 %), it can be noted that the proportion of women who had had a previous induced abortion was highest among the divorced/separated/widowed (34 %).

**Table 1:** Socio-demographic characteristics of women seeking post-abortion care by history of induced abortion

Variable	First-time abortion [%]	Repeat abortion [%]	Sample size [Unweighted]
Age category			*p = 0.005*
10–19 years	92.8	7.2	174
20–24 years	78.3	21.7	240
25 + years	83.3	16.7	353
Missing	100.0	0.0	2
Residence			*p < 0.1*
Urban	78.9	21.1	421
Rural	88.6	11.4	346
Missing	37.9	62.1	2
Marital status			*p < 0.05*
Never married	87.8	12.2	406
Married/Living together	87.0	13.0	273
Separated/Divorced/widowed	66.3	33.7	88
Missing	100.0	0.0	2
Education			*P = 0.930*
No education	83.3	16.7	20
Primary	85.5	14.5	225
Secondary	82.8	17.2	331
Post-secondary	86.1	13.9	191
Missing	100.0	0.0	2
Total	84.4	15.6	
*N*	659	110	769

Table shows weighted proportions and unweighted sample sizes

Chi-square tests were used to test the significance in differences

The proportions of women seeking treatment for first time induced abortion or women who had had a prior induced abortion by reproductive health characteristics are presented in Table 2. Notable significant differences were only observed by contraceptive use at the time of conception. Higher proportions of women seeking care after a repeat induced abortion were observed among women who were on traditional methods of contraception (43 %) followed by those on short-acting methods (22 %) and least among those on long-acting methods (7 %). Though not statistically significant, higher proportions of women seeking care after a repeat induced abortion were observed among women who were uncertain about the wantedness status of their pregnancies (31 %) and those who did not want the pregnancy at the time of conception (18 %). Nine percent of women who wanted the current pregnancy at the time of conception reported having had a prior induced abortion.

**Table 2:** Reproductive health characteristics of women seeking post-abortion care by history of induced abortion

Variable	First-time abortion [%]	Repeat abortion [%]		Sample size [Unweighted]
Previous live births			*p < 0.399*	
None	86.9	13.1		367
1–2 births	80.0	20.0		239
3 or more births	85.3	14.7		162
Missing	100.0	0.0		1
Pregnancy wantedness			*p < 0.250*	
Wanted then	90.7	9.3		64
Wanted later	87.8	12.2		239
Did not want	82.5	17.5		433
Don’t know/Missing	69.0	31.0		33
Gestational age			*P = 0.588*	
< =12 weeks	84.7	15.3		486
> 12 weeks	83.3	16.7		278
Unknown/indeterminate	100.0	0.0		5
Contraceptive use at the time of conception^a^			*p* < 0.01	
No method	89.2	10.8		461
Short-acting method (SACM)	78.4	21.6		267
Long acting method (LACM)	92.8	7.2		15
Traditional method	56.6	43.4		26
Total	84.4	15.6		
*N*	659	110		769

Table shows weighted proportions and unweighted sample size

Chi-square tests were used to test the significance in differences

^a^Short-acting methods include pills, injections, male and female condoms, diaphragm, foam/jelly, patch and emergency contraception; Long-acting methods include implants, female and male sterilization, IUD; Traditional methods include rhythm, lactational amenorrhea, and withdrawal

Table 3 presents the results of the age-standardized models analyzing the factors associated with repeat induced abortion. Variables with significant effects include: marital status, education, previous live births, pregnancy wantedness, and contraceptive use at the time of conception. Women who were separated/divorced/widowed were six times more likely to have had a prior abortion compared to single women who had never been married (OR = 6.804; *p* = 0.000). Women with unwanted pregnancies (OR = 0.534; *p* = 0.021) and those who wanted the pregnancy later (OR = 0.347; *p* = 0.025) were less likely to have had a prior abortion compared to those whose pregnancy was wanted. Compared to women with no education, women with education were less likely to have had a prior abortion (OR = 0.284, *p* = 0.001 (primary); OR = 0.336, *p* = 0.010 (secondary); OR = 0.278, *p* = 0.002 (post-secondary)) while women with 1–2 previous live births were 2 times more likely (OR = 2.104; *p* = 0.012 to have had a prior abortion compared to women who had no previous live births. Finally, compared to women not using any contraceptive method at the time of conception, women on traditional methods of contraception and women on short-acting methods (although not significant) were nine times (OR = 9.474; *p* = 0.004) and two times (OR = 2.308; *p* = 0.054) respectively more likely to have had a prior abortion compared to women not using any contraceptive method at the time of conception.

**Table 3:** Factors associated with repeat abortion; results from aged-standardized regression models

Variable	Odds ratio (OR)	Level of significance (*p* value)	95 % Confidence interval (CI)
Residence			
Rural (ref.)	1.000		
Urban	1.696	0.219	0.729–3.949
Marital status			
Never married (ref.)	1.000		
Married/Living together	1.748	0.123	0.858–3.559
Separated/Divorced/Widowed	6.804	**0.000**	2.778–16.663
Education			
No education (ref.)	1.000		
Primary	0.284	**0.001**	0.141–0.574
Secondary	0.336	**0.010**	0.148–0.765
Post-secondary	0.278	**0.002**	0.125–0.620
Previous live births			
None (ref.)	1.000		
1–2 births	2.104	**0.012**	1.182–3.745
3 or more births	2.503	0.086	0.878–7.134
Pregnancy wantedness			
Wanted then (ref.)	1.000		
Wanted later	0.347	**0.025**	0.138–0.874
Did not want	0.534	**0.021**	0.313–0.910
Unsure/don’t know	1.185	0.768	0.381–3.682
Gestation age			
< =12 weeks (ref.)	1.000		
> 12 weeks	0.885	0.623	0.543–1.442
Contraception^a^			
Not using (ref.)	1.000		
Short-acting method (SACM)	2.308	0.054	0.984–5.415
Long acting method (LACM)	1.068	0.934	0.223–5.123
Traditional method	9.474	**0.004**	2.043–43.934

*Ref.* reference category

Bold and italic *p*-values represent *p* < 0.05

^a^Short-acting methods include pills, injections, male and female condoms, diaphragm, foam/jelly patch and emergency contraception; Long-acting methods include implants, female and male sterilization and IUD; Traditional methods include rhythm, lactational amenorrhea, and withdrawal

## Discussion

Despite the illegality of abortion in Kenya, previous research has shown a relatively high magnitude of unsafe abortions [7, 23]. With the increasing incidence of induced abortion, it is highly likely that the rate of repeat induced abortions will also increase and as our results show, the higher proportion of repeat abortions among women aged 20–24 than 25 and above suggests that the abortion rate is increasing in Kenya. Repeat induced abortions can be prevented if the associated factors are known, i.e. if women with high likelihood of a repeat abortion can be identified and targeted for specific interventions. This study investigated the factors associated with repeat induced abortions among women seeking care for complications from induced abortions in a nationally-representative sample of health facilities. Findings indicate that a minimum of 16 % of women seeking post abortion care services in public and private health facilities in Kenya in 2012 had had a prior abortion. Women who were formerly in marital unions (divorced/separated/widowed), those using traditional methods of contraception, and those with 1 – 2 prior births were more likely to have had a prior induced abortion. On the other hand, women with primary, secondary or post-secondary level of education and those whose index pregnancy was unwanted or wanted later were associated with a lower likelihood of having had a prior induced abortion.

Contrary to findings that show high proportions of women having repeat abortions being in marital unions [24] our findings show that separated/divorced/widowed (34 %) were seeking care for a second or later abortion compared to 13 % among the married women and 12 % among the never married women. A study carried out in Canada had similar results and showed that women who were previously married and women in common-law relationships had an above average proportion of repeat abortions [25]

Similar to other studies in Finland [11] and Estonia [26], our findings indicated striking differences in the rate of repeat abortion between women using short-acting and traditional methods and those using long-acting methods with the former associated with the highest rate of repeat abortions. As regards to the association with live births, our findings echo the results of a study carried out in the United States of America where women in higher parity were more likely to have repeat abortions [27]. In their study, Jones et al. found that women having repeat abortions were not only likely to have had prior births, but were more likely to have had three or more prior births [27]. Another study carried out in Nepal had similar findings [5].

This study indicates that over 17 % of women who had an unwanted pregnancy and 12 % who had a mistimed (not wanted then) pregnancy had had a previous induced abortion. However, these results are contradicted by our regression results which, although not supported by existing literature, shows that women who had unintended pregnancies (‘wanted later’ and ‘did not want then’) at the time of conception were less likely to report having a prior abortion compared to those who had wanted the pregnancy then. Extant literature shows that unwanted pregnancies at the time of conception are more likely to end up in abortion compared with wanted pregnancies. However, circumstances that women experience in the course of pregnancy may lead a woman to abort even a wanted pregnancy. For example, the status of the relationship with partner may have changed after the pregnancy occurs, women may experience increased pressure from partners to end a pregnancy or women in abusive relationships may abort a pregnancy [28–30] to avoid bringing a child into an abusive relationship. Most studies have associated unwanted pregnancy with contraceptive non-use at the time of conception [14, 16, 17] and contraceptive method failure due to inefficient use as well method ineffectiveness [5, 16, 31, 32]. A study by Cheng et al. among a sample of women seeking an abortion in China indicated that about 70 % of women who had had more than one abortion did not use a contraceptive method at their first sexual intercourse after the procedure. The study further found that while almost 48 % of the current pregnancies were associated with contraceptive non-use, 52 % were attributed to contraceptive failure [16].

## Conclusions

According to the Programme of Action adopted at the International Conference on Population and Development, Cairo (1994) governments and relevant intergovernmental and non-governmental organizations are urged to strengthen their commitment to women’s health, to deal with the health impact of unsafe abortion as a major public health concern and to reduce the recourse to abortion through expanded and improved family planning services. Prevention of unwanted pregnancies must always be given the highest priority and every attempt should be made to eliminate the need for abortion. Women who have unwanted pregnancies should have ready access to reliable information and compassionate counseling. Post-abortion counseling, education and family planning services should be offered promptly, which will also help to avoid repeat abortions. (Paragraph 8.25) [26].

Efficient and correct use of modern contraceptive methods has real potential to decrease unintended pregnancy and repeat abortion. Counseling and commodities should be accessible to all women, including those who are young. There is also need to enhance youth friendly health care facilities and youth friendly health practitioners who are able to counsel the young people and provide information and education on contraceptive use. The findings of this study point to the fact that family planning programs have a long way to go towards addressing issues of unintended pregnancies in the Kenyan context. They also point to the need to address the reasons why women with first time induced abortion do not have the necessary information to prevent unintended pregnancies and further induced abortions. Many health facilities in Kenya do not integrate post abortion services and family planning services and referral systems are not well designed enough to avoid losing women in need of contraception in between. The Magnitude and Incidence of Unsafe Abortion Project data indicated for instance that 48 % of women seeking post abortion care services for first time abortions did not receive contraception methods at discharge. The corresponding percentage was 22 % among women seeking post abortion care services for repeat abortions. Comprehensive and integrated information on contraception and preferably on long term acting and permanent method should be systematically offered to young and older women seeking for post abortion services in all public and private facilities in Kenya. Facilities offering post-abortion care services should encourage women to use modern methods of contraception and especially long-acting methods and also discourage use of traditional methods as they have proven to be ineffective.

### Limitations

The rate of previous induced abortion was based on women’s self-reporting and hence what was reported could be termed as a minimum estimate. The illegality of abortion in Kenya further heightens the risk of underreporting. Moreover, data were collected only from women seeking help for complicated abortions in health facilities. This limitation also considers the availability of medical abortion services which can be obtained over the counter. Majority of women undergoing medical abortion may not experience complications related to unsafe abortion hence not presenting for post abortion services at heath facilities.

## Footnotes

1 The WHO report on “Unsafe Abortion: Global and regional estimates of the incidence of unsafe abortion and associated mortality in 2008” defines the Eastern Africa region as Burundi, Comoros, Djibouti, Eritrea, Ethiopia, Kenya, Madagascar, Malawi, Mauritius, Mayotte, Mozambique, Reunion, Rwanda, Somalia, Uganda, United Republic of Tanzania, Zambia and Zimbabwe.
